# Editorial: Harnessing genebanks: High-throughput phenotyping and genotyping of crop wild relatives and landraces

**DOI:** 10.3389/fpls.2023.1149469

**Published:** 2023-03-10

**Authors:** Andrés J. Cortés, Jinyoung Y. Barnaby

**Affiliations:** ^1^ Corporación Colombiana de Investigación Agropecuaria – AGROSAVIA, C.I. La Selva, Rionegro, Colombia; ^2^ U.S. Department of Agriculture, U.S. National Arboretum, Floral and Nursery Plants Research Unit, Beltsville, MD, United States

**Keywords:** germplasm, *ex situ* conservation, food security, pre-breeding, exotic variation, elite varieties, polygenic variation, adaptation

## Introduction

Worldwide genebanks hold phenotypic and genetic novelty useful to increase yield, crop adaptability, and agrobiodiversity (Tanksley and McCouch, 1997) while buffering crop genetic erosion (Khoury et al., 2021). However, new strategies for genebank utilization must be empowered in order to meet increasing global food demand (McCouch, 2013; Bohra et al., 2021) with crop alternatives resilient to climate change, sustainable to the environment and the biodiversity, and profitable for communities (Scherer et al., 2020). Therefore, in order to contribute filling this gap on genebank mining, this Research Topic compiles recent developments able to speed up crop improvement processes by leveraging high-throughput phenotyping and genotyping of crop wild relatives (CWR) and landraces (Singh et al., 2022). As discussed in the next section, the amassed works innovate different steps of genebank characterization, utilization, and allelic deployment, including germplasm identification, conservation, pre-breeding screening for genepool diversity and associated markers, and introgression breeding.

## Mining and unlocking the hidden value of CWR and landraces

Crop wild relatives are feasible sources for novel genetic and phenotypic variation (Bohra et al., 2021), as exemplified by Inagaki et al. The team demonstrated that a rice (*Oryza sativa* L.) near-isogenic line (NIL) that carries a genomic segment on chromosome seven from a Thai *O. rufipogon* CWR, narrowed by marker-assisted selection from a BC_4_F_2_ generation, is able to capture an optimum spectrum of plant shapes for sunlight receiving efficiency, which in turn maximizes vegetative growth and weed suppression. The historical crop-wild transition and genetic interchange during domestication also reinforces the value of wild genepools, as shown by Yuan et al. The team recovered loci responsible for seed coat color pattern during domestication of wild soybean [*Glycine max* (L.) Merr.] through whole-genome re-sequencing of 276 F_10_ recombinant inbred lines (RILs) derived from a cross between cultivated and wild soybean accessions. Phenotypic variations from CWR may also be retained in the offspring between cultivated and wild genepools, as demonstrated by Li et al. (2021) Specifically, they captured 31 agronomic-relevant QTL and allelic variation (*e.g.*, for grain formation and length) from weedy rice (*Oryza sativa* f. *spontanea*) *via* whole-genome sequencing of a 199 F_2_ population obtained by crossing a weedy rice with low heterozygosity and a cultivated rice variety. These results by Inagaki et al. (2021), Yuan et al., and Li et al. (2021) exemplify the utilization of wild genetic resources to increase the genetic base of cultivated crops for sustainable and resilient agricultural production.

Landraces, having been selected under subsistence agricultural environments, also hold a broad representation of useful natural variation (McCouch, 2004). For instance, Osterman et al. used LASSO models to link environmental variation with adaptive genetic diversity in red clover (*Trifolium pratense* L.), a perennial temperate forage legume, through genotyping 382 accessions from Scandinavia, including landraces and CWR, with 661 single nucleotide polymorphism (SNP) markers derived from seqSNP-targeted sequencing. The genomic landscape of genetic variation was also unveiled by Mulugeta et al. The authors used a diversity panel of durum wheat (*Triticum durum* Desf.) collected from the Ethiopian highlands, and identified 44 genomic regions associated with grain yield and related traits using last-generation (*i.e.*, FarmCPU) genome-wide association study (GWAS) models that inputted 10,045 SNP markers derived from the 25k Illumina wheat SNP array. In order to further explore isolated pockets of cryptic agrobiodiversity in Ethiopia, Enyew et al. and Gebeyehu et al. respectively examined the patterns of genetic diversity in sorghum [*Sorghum bicolor* (L.) Moench] and noug (*Guizotia abyssinica*), an outcrossing edible oilseed crop. The former team genotyped a total of 359 sorghum individuals, comprising 24 landrace accessions, with 3,001 gene-based single nucleotide polymorphism (SNP) markers, and found that sorghum accessions with bent peduncles exhibited more genetic variation than those with erect peduncles (Enyew et al.). The latter team performed RNA-Seq based transcriptome sequencing of 30 noug genotypes (Gebeyehu et al.), and predictably captured greater genetic similarity among self-compatible genotypes compared to self-incompatible accessions. Finally, Nuraga et al. introduced enset (*Ensete ventricosum*), a multipurpose crop grown in southern Ethiopia for human food, animal feed, and fiber. They demonstrated that enset landraces were not genetically differentiated based on their use-value (*i.e.*, medicinal *vs.* non-medicinal landraces) through genotyping 51 accessions with 15 simple sequence repeat (SSR) markers. Altogether, the works by Osterman et al. (2022), Enyew et al. (2022), Gebeyehu et al., and Nuraga et al. demonstrate the potential of landraces as sources of genetic innovation for agrobiodiversity.

The latter study by Nuraga et al. also illustrates how CWR and landraces are being used for exotic non-conventional purposes (Von Wettberg et al., 2020). Following Nuraga et al., medicinal applications were also addressed in the work by Cui el al., using Gardenia (*Gardenia jasminoides* Ellis), a Chinese perennial shrub from the Rubiaceae family with edible flowers and medicinal fruits. The authors conducted syntenic analyses with model species within the Rubiaceae family (*i.e.*, *Coffea arabica*, *C. canephora*, and *Ophiorrhiza pumila*), and discovered 18 and 31 QTL associated with growth- and leaf-related traits, respectively, by genotyping 200 F_1_ hybrids with 4,249 genotyping by sequencing (GBS)-derived SNP markers. The couple of studies by Nuraga et al. and Cui et al. provide fundamental knowledge that enables further exploration of alternative uses of CWR and landraces (Wu et al., 2022).

Meanwhile, hybridization has played a key role in bidirectional adaptive introgression (Abbott et al., 2013) as well as hybrid breeding within crop-wild complexes (Cortés et al., 2022a). For example, Volk et al. (2022) explored the signatures of admixture from the cultivated apple [*Malus domestica* (Suckow) Borkh] into the progenitor species *M. sieversii* (Ledeb.) M. Roem., *M. orientalis* Uglitzk., and *M. sylvestris* (L.) Mill. (Cornille et al., 2014) by genotyping 463 accessions using the 20K apple SNP array. On the other hand, Conejo-Rodriguez et al. leveraged hybrid phenotyping for pre-breeding in beans (*Phaseolus* spp.). They proposed an innovative pipeline to determine parental phenomic proportions in hybrids, and validated it using interspecific crosses derived from common bean (*P. vulgaris* L.), tepary bean (*P. acutifolius* A. Gray), and its wild relative *P. parvifolius* Freytag. The latter two served as donors of heat and drought tolerance (tepary, Buitrago-Bitar et al., 2021), and resistance to common bacterial blight (CBB), respectively (Conejo-Rodriguez et al.). Both studies by Volk et al. (2022) and Conejo-Rodriguez et al. (2022) highlight introgression breeding as a still valid alternative to bridge historical barriers between crops and their wild genepools (Labroo et al., 2021).

An additional study harnessing modern phenomic ‘big data’ tools in diverse genepools was reported by Restrepo-Montoya et al. They utilized qualitative descriptors to curate and catalogue cotton (*Gossypium* spp.) germplasm encompassing an impressive dataset of 1,616 observations between 2011 and 2019 on 7,941 unique accessions and 50 species. The last two pre-breeding phenotyping efforts carried out by Conejo Rodriguez et al. and Restrepo-Montoya et al. enable designing and targeting downstream goals in breeding by defining trait families, categorizing germplasm diversity, and pinpointing exotic outlier accessions.

Although great advancement has been made in the field of phenotyping, matching the exponential achievement from the genomic arena still requires more innovative analytical tools. Candidate platforms should be capable to merge and interpret complex multi-dimensional datasets embracing diversity from CWR and landraces. To this end, the review by Tirnaz et al. (2022) envisioned novel avenues to utilize genebank resources, for instance *via de novo* domestication, genome editing, and speed breeding. This review further advocated for computational (*e.g.*, machine learning) approaches suitable for speeding up the incorporation of exotic genepools into breeding programs to improve crop production, adaptability and sustainability (Tirnaz et al.).

## A roadmap to harness genebanks

**Figure 1:**
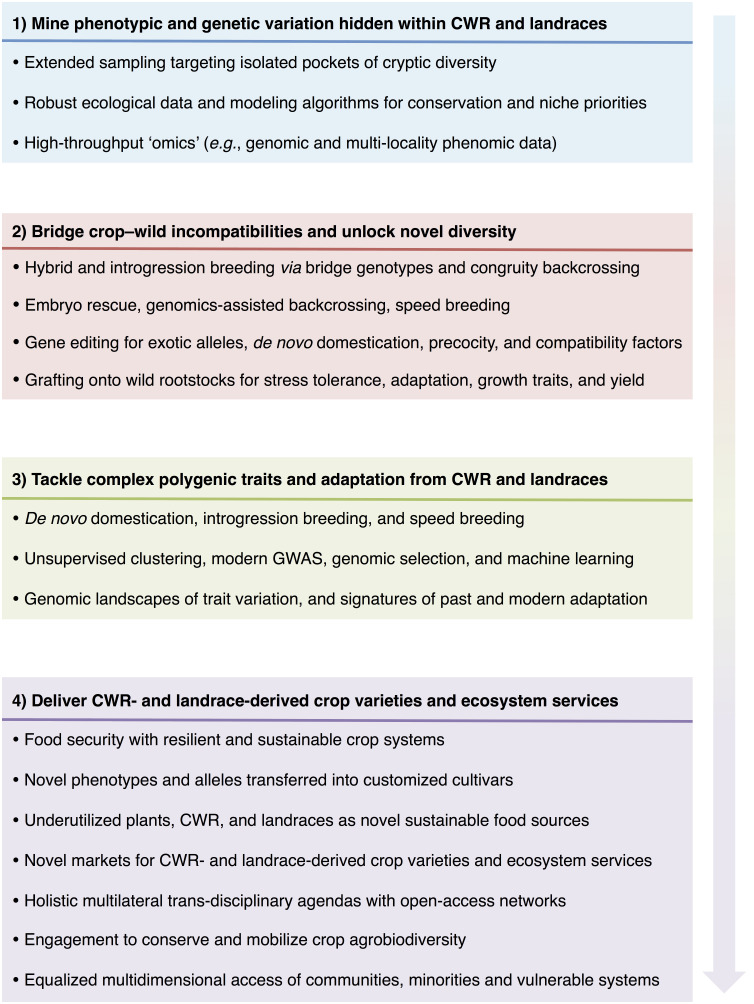
Schematic roadmap to harness genebanks. Each box marks pivotal research avenues (with the corresponding recommendations within) worth considering for mining, unlocking and utilizing phenotypic and genetic novelty hidden within crop wild relatives (CWR) and landraces. Ultimately, achieving food security with resilient and sustainable agrifood systems capable to cope with climate change, biodiversity loss, and increased pollution would require nature-based solutions for climate-smart alimentary schemes inspired in underutilized plant species, CWR, and landraces.

Crop wild relatives and landraces stored at genebanks harbor unique variation that may benefit food security, sustainability (Tanksley and McCouch, 1997) and resilient climate change adaptation (Renzi et al., 2022). Yet, their factual utilization has been hampered by poor characterizations, genetic incompatibilities, and polygenic variance (Coyne et al., 2020). Therefore, an improved roadmap to address these bottlenecks with modern technologies is a prerequisite for their effective utilization (**Figure 1**). A first pivotal research avenue worth considering is mining phenotypic and genetic variation hidden within CWR and landraces (Singh et al., 2022) through extended sampling targeting isolated pockets of cryptic diversity (Ramirez-Villegas et al., 2020), robust ecological data curation (Waldvogel et al., 2020b) [*e.g.*, targeting specific abiotic stresses such as drought (Cortés and Blair, 2018) and heat tolerance (López-Hernández and Cortés)], dense linkage disequilibrium (LD) guided genomic characterizations (Blair et al., 2018), and geographic-wide agronomical (Osterman et al. 2022) and physiological (Conejo-Rodriguez et al.) trials across diverse germplasm and environments [recently referred to as enviromics (Costa-Neto and Fritsche-Neto, 2021; Crossa et al., 2021; Resende et al., 2021)].

Second, bridging crop–wild incompatibilities and unlocking novel diversity may rely on hybrid breeding *via* bridge genotypes (Conejo-Rodriguez et al. 2022), and introgression breeding (Migicovsky and Myles, 2017) from wild genepools into cultivars [*e.g.*, Inagaki et al. (2021), Yuan et al. (2022), and Li et al. (2021)], and viceversa (Volk et al.). To overcome genotype incompatibility and embryo abortion issues, typically found in simpler backcrosses, pre-breeding schemes could be advanced into multiple alternating backcrosses between exotic donors and elite genepools (Burbano-Erazo et al., 2021), as in classical congruity backcrossing (Muñoz et al., 2003). Other promising alternatives to achieve a more optimal retention of the exotic phenotype include: embryo rescue, genomic-assisted backcrossing (Migicovsky and Myles, 2017), speed breeding (Watson et al., 2018; Alves et al., 2020; Bohra et al., 2021), tissue-specific gene editing [*e.g.*, for exotic alleles (Martignago et al., 2019), *de novo* domestication (Lemmon et al., 2018), precocious flowering time, and compatibility factors (Fritsche et al., 2018)], and grafting with wild rootstocks [*e.g.*, for biotic and abiotic stress tolerance (Warschefsky et al., 2016), adaptation to soil toxicity (Fernández-Paz et al., 2021), growth traits (Cañas-Gutiérrez et al., 2022), and yield (Reyes-Herrera et al., 2020)]. Merging these strategies could facilitate even quicker transitions from the wild genepools compared to classical inter-specific and crop-wild controlled pollination.

As a third step, major challenges to utilize CWR and landraces for improvement of complex polygenic adaptive traits (Renzi et al., 2022) can be tackled by linking last-generation experimental setups [*e.g.*, diversifying selection (McCouch, 2004), introgression breeding (Muñoz et al., 2003; Burgarella et al., 2019), speed breeding (Watson et al., 2018; Alves et al., 2020; Kumar et al., 2020; Varshney et al., 2021b), *de novo* domestication (Fernie and Yan, 2019), and genome editing (Lemmon et al., 2018)] with modern analytical developments [*e.g.*, last-generation genetic clustering (Enyew et al., 2022; Gebeyehu et al., 2022; Nuraga et al., 2022) and mapping Mulugeta et al. 2023, predictive breeding (Ahmar et al., 2021), genomic-informed selection (Desta and Ortiz, 2014; Crossa et al., 2017), and machine learning (Varshney, 2021; Tirnaz et al., 2022)]. Such trans-disciplinary approaches (Tirnaz et al., 2022) will enable better reconstructions of the genomic landscapes (Ellegren and Wolf, 2017; Barnaby et al., 2020; Tong and Nikoloski, 2021) of trait variation (Barnaby et al., 2022; Li et al.; Yuan et al.), and the selection signatures of past (Cortés et al., 2020; Waldvogel et al., 2020) and modern adaptation (Cortés et al., 2022b; Osterman et al., 2022). Retrieving complex polygenic interactions across the genomic architectures of quantitative (Boyle et al., 2017) and adaptive traits (Barrett and Hoekstra, 2011; Barghi et al., 2020) is a prerequisite to pinpoint exotic alleles enclosed within CWR and landraces (Cortés et al., 2012), and speed up the utilization of this natural variation as part of conservation and pre-breeding programs (Migicovsky and Myles, 2017).

Fourth, novel approaches need to de defined in order to leverage CWR and landraces (Varshney et al., 2021b; Tirnaz et al.). After all, boosting crop adaptability, sustainability, and yield in the face of changing climate, biodiversity loss, and increased pollution requires multilateral trans-disciplinary efforts. Various actors, including producers, scientist and transfer officers, decision makers, funding bodies and marketers should converge to plan and implement innovative strategies capable to meet future global food demands, while facing enlarged threats from abiotic (Blair et al., 2016; Cortés and López-Hernández, 2021) and biotic (Guevara-Escudero et al., 2021) pressures. Some of this pioneering strategies may include (1) prospection of genotypes candidate for exotic alleles (Migicovsky and Myles, 2017), ancient cultivars (Atchison et al., 2016; Thapa et al., 2021), and novel crops (Von Wettberg et al., 2020; Cui et al., 2022; Nuraga et al.); (2) multidimensional ‘big data’ compilation and management from the scientific, climatic and marketing arenas (Tirnaz et al., 2022) to identify conservation priorities (Castaneda-Alvarez et al., 2016), novel markets and value chains (Smale and Jamora, 2020), and (3) innovation for the production, transformation, delivery, commercialization and utilization of CWR- and landrace-derived crop varieties (McCouch, 2013) and ecosystem services (Tyack et al., 2020).

## Perspectives

Further studies and implementation practices are urgently required to better harness high-throughput phenotyping and genotyping of CWR and landraces at genebanks. Ultimately, CWR and landraces can be utilized as resources to effectively explore phenotypic and genetic variants associated with innovative phenotypes, and to transfer those traits into customized cultivars (Varshney et al., 2021a). Alternatively, CWR and landraces may themselves be adopted as novel food sources (Von Wettberg et al., 2020). For these strategies to succeed, holistic multilateral and trans-disciplinary agendas should also prioritize an open-access networks approach (Spindel and McCouch, 2016) for mobilizing crop biodiversity (McCouch et al., 2016). On balance, embracing food security with resilient and sustainable crop systems requires equalizing multidimensional access of communities, minorities and vulnerable systems, including farmers from developing countries, women in agriculture, early-career researchers, global South-South partners, and orphan crops. Only then, underutilized plants, CWR and landraces would play an active role as nature-based solutions for agrifood systems.

## Author contributions

AC conceived the Research Topic with insights from JB. Both authors made substantial contributions in preparing, editing and reviewing the contents of the Research Topic on “*Harnessing Genebanks: High-Throughput Phenotyping and Genotyping of CWR and Landraces*”. AC wrote a first version of the editorial, later edited by JB. Both approved it for publication.
